# Ubique Gentium?

**Published:** 1870-05

**Authors:** 


### Editorial.

*Ubique Gentium?*

Mr. Leckey, the author of the masterly "History of European Morals"—a work which every thinking man should not only read but ponder well—a work which may rank among the highest productions of the cultured intellect, of the last quarter of a century, enunciates with regard to the medical profession these views, which are probably to be accepted as the deliberate sense of very many, if not of the majority, of the thinking men, the men of culture, (non-professional) of the present time.

"Of all the great branches of human knowledge, medicine is that in which the accomplished results are most obviously imperfect and provisional, in which the field of unrealized possibilities, is most extensive, and from which, if the human mind were directed to it, as it has been during the past century to inventions, and especially to overcoming space, the most splendid results might be expected. Our almost absolute ignorance of the causes of some of the most fatal diseases, and the empirical nature of nearly all our best medical treatment, have been often recognized. The medicine of inhalation is still in its infancy, and yet it is by inhalation that nature produces most of her diseases, and effects most of her cures. The medical powers of electricity, which of all known agencies bears most resemblance to life, are almost unexplored. The discovery of anæsthetics has in our own day opened out a field of inestimable importance, and the proved possibility, under certain conditions, of governing by external suggestions the whole current of the feelings and emotions, may possibly contribute yet further to the alleviation of suffering, and perhaps to that Euthanasia which Bacon proposed to physicians as the end of their art."

Mr. Leckey discusses then the relations between our physical and moral nature, and suggests that, with the complete information it is possible to acquire, we may treat the many varieties of moral disease as systematically as we now treat physical diseases.

Sir William Hamilton, another eminent thinker of the present time, curtly characterizes the history of medicine as a "History of Variations."

Medical scholars, themselves, in looking over our records, have not infrequently made use of indignant and almost despairing sentences—even harsh and coarse objurgations, at the fickleness of opinion, the contradictory statements, the multiform hypotheses, the myriad "remedies," the acrimonious disputes, the vehement sarcasms and loud ridicule which have chased each other through all the bewildering mazes of professional history. Of course these hasty expressions, having little more to do with the argument than exclamation points in punctuation of a book, are greedily caught up by the advocates of exclusive and dogmatic "new systems of practice;" and even philosophical and cultivated minds are apt to attribute to them an importance and weight utterly beyond their real value. Montaigne, who had "the stone," and found no relief from the physicians of his age, railed at the whole fraternity, and every little essayist and popular lecture maker, since, has imitated this great master of the sixteenth century, forgetting that the world is three hundred years older. Montaigne recognized the mistakes into which the physician must almost of necessity fall.

"He has need of too many parts, considerations, and circumstances rightly to adjust his design; he must know the sick person's complection, his temperature, his humors, inclinations, actions, nay, his very thought and imaginations; he must be assured of the external circumstances, of the nature of the place, the quality of the air, and season, the situation of the planets, and their influences; he must know in the disease, the causes, prognostics, affections, and critical days; in the drugs, the weight, the power of working, the country, figure, age, and dispensation; and he must know how rightly to proportion and mix all these together, to beget a just and perfect symmetry; wherein if there be the least error, if amongst so many springs, there be but any one out of order, 'tis enough to destroy us. God knows of how great difficulty most of these things are to be understood."

But the trouble is not alone of what physicians should, but do not, know, for as old Thomas Browne wrote:

"For, what is worse, knowledge is made by oblivion; and to purchase a clear and warrantable body of Truth, we must forget and part with much we know." * * "Wise men cannot but know, that Arts and Learning want this expurgation; and if the course of truth bee permitted unto its selfe, like that of Time and uncorrected computations, it cannot escape many errors, which duration still enlargeth."

And these latter hit exactly the pith and marrow of the subject. So long as diseases were considered as entities, as devils to be cast out, and symptoms as the manifest strivings of these devils with the possessed organ or body, just so long would continue the search for mysteriously operating agents, or charms, or as the later word to express the same idea is, specifics. Even LORD BACON fell into this identical error, notwithstanding his usually clear insight of the methods of discovering truth, as is instanced in the often quoted sentence; complaining that physicians neglect efforts to discover particular remedies having a peculiar and especial relation to particular symptoms and diseases. Mr. Leckey, by his reference to inhalations, electricity and anæsthetics, in the passage quoted, evidently looks for the same thing, only he wants us to discover for him some specifics for moral diseases—some "Morrison's Pill" (as Carlyle has it) for disorders of the psychical part, both of individuals and the commonwealth. The thought was anticipated by Macbeth—which see, for there it is in Scene III, Act V.

BROUSSAIS characterized this idea in medicine as *Ontology*, and his explanation, half a century ago, shows now, as then, why much of medicine, so called, remains in darkness and uncertainty.

"The group of symptoms which are given as diseases, without referring them to the organs, on which they depend, or referring them to the organs without having well determined the nature of the physiological aberrations of these last, are metaphysical abstractions, which do not represent a constant, invariable morbid state, of which we can be sure of finding the model in nature; they are then factitious existences, and all who study medicine by this method, are *ontologists*.

"To consider these factitious morbid existences as injurious powers, which, act upon the organs, and modify them by producing such or such a disorder, is to mistake effects for causes; is ontology. To consider the succession of symptoms which have been observed, as the necessary and invariable progress of the disease, and to derive from them characters essential to their diagnosis, and consequently to their treatment, is to create a factitious being; since the affections of organs are different, according to the modifying powers which act upon them; it is placing ourselves in the impossibility of treating the disease before its termination, without contradicting our own principles. This is always ontology."

About everybody in these days will disclaim ontological views with regard to disease, but its nomenclature contaminates the books, and infests the language of professional life. The physician of highest culture brought face to face with a sick man, will, involuntarily, perhaps, nevertheless in fact, look upon the disease as a personality, to be dealt with as an enemy. The whole enginery of formularies reeks with it.

From a pamphlet before us, the valedictory address at Jefferson Medical College, March, 1870, by J. AITKIN MEIGS, M.D., Prof., etc., we extract these sentences:

"To the reflecting mind it is evident that medicine is now passing through a chaotic phase in its own career. The day of blind obedience to authority is at an end. No asserted fact, no theory, however plausible, finds its way to acceptance on account of the great name attached to it, but on the contrary, it is immediately tried in the crucible of experiment, observation, and induction. But medicine, in becoming scientific, has grown sceptical. The twin brothers Doubt and Disbelief are even now pulling up, and with no gentle hand, the ancient landmarks all around us. The iconoclasts are at work. Opinions and conclusions are being sifted so fiercely that we at length stand face to face with the danger of denying altogether the accumulated experience of our fathers."

Language like this could not have been used without imperiling the professional *status* twenty years ago, but it is central truth, and the writer, in current phrase, strikes the hard-pan or bed rock, in speaking of the reconstruction of the philosophy of medicine and placing it upon a sure scientific basis.

"This basis, it is now beginning to be seen, is to be made up of the accumulated facts relating to the chemical circulation of matter, the conservation of energy, and the development of organic forms by natural selection."

We are moving on — falling into the line of the world's progress, by no pitiful discoveries of specifics, big or little; no dubious inhalations, electrical wonders or anæsthetic stupefaction, such as Mr. Leckey hints to be the guide-boards of advance.

We do not argue here, yet do not in anywise admit that medicine has lagged behind other sciences, whether of locomotion or other. Hygiene and philosophical prophylaxis did more to ensure success in the late war than even railroads or improved guns.

We are able to say positively, to malignant epidemics, which once swept away population by the millions: Thus far shalt thou go and no farther! Considering the nature of the subject, we are willing to be tested by similar methods to those which Mr. Lecky applies to the particulars of general progress.

We concede the defects both of our Science and Practice, but we proudly claim to belong to a profession whose advancement challenges the rivalry of any other under the sunlight.

